# A dominantly-inherited Behcet-like disorder caused by haploinsufficiency of the TNFAIP3/A20 protein

**DOI:** 10.1186/1546-0096-13-S1-O71

**Published:** 2015-09-28

**Authors:** Q Zhou, H Wang, J Chae, D Yang, E Demirkaya, M Stoffels, M Takeuchi, C Chen, A Ombrello, D Schwartz, P Hoffmann, D Stone, R Laxer, AV Royen-Kerkhof, S Ozen, M Gadina, D Kastner, I Aksentijevich

**Affiliations:** 1 NHGRI/NIH, Bethesda, USA; 2 NHLBI/NIH, Bethesda, USA; 3 Institute of Health Sciences, Ankara, Turkey; 4 NIAMS/NIH, Bethesda, USA; 5 The Hospital for Sick Children, Toronto, Canada; 6 Universitair Medisch Centrum Utrecht, Utrecht, Netherlands; 7 Hacettepe University, Ankara, Turkey

## Introduction

*TNFAIP3* encodes the anti-inflammatory A20 protein that functions as a potent negative regulator of NFκB signaling and the NLRP3 inflammasome. Low penetrance common variants of *TNFAIP3* have been associated with a number of autoimmune diseases. Here we report 5 high penetrance dominantly-inherited frameshift and nonsense *TNFAIP3* mutations in 11 patients with early-onset systemic inflammation, arthralgia/arthritis, oral and genital ulcers, and ocular inflammation.

## Objectives

To identify a possible genetic cause of dominantly-inherited early-onset systemic inflammatory disease.

## Patients and methods

We performed exome sequencing in 3 families, candidate gene screening in 2 families, and targeted sequencing of 384 Turkish and 384 Japanese patients. We utilized immunoblotting, cytokine profiling, immunostaining, immunofluorescence, real-time PCR, and flow cytometry to study abnormalities in patients’ immune cells.

## Results

Four *TNFAIP3* mutations were located in the N-terminal OTU domain of A20 and generated truncated proteins of similar length, while the fifth mutation was a truncating frameshift in the more C-terminal ZnF4 domain. Targeted sequencing of *TNFAIP3* in Turkish and Japanese GWAS cohorts with Behcet’ disease identified one patient with a novel frameshift mutation in the OTU domain. None of the mutations were found in any public database. Expression of A20 was reduced in patients’ PBMCs and fibroblasts relative to healthy controls, and the mutant truncated proteins were not detectable by Western blots. Overexpression of wild type (wt) A20 inhibited TNF-α-induced NF-κB activity and removed K63 ubiquitin chains from RIP1, NEMO, and TRAF6, whereas A20 mutants did not. Co-expression of wt and mutant A20 demonstrated haploinsufficiency of the mutant protein rather than a dominant negative effect. In leukocytes from patients possessing A20 mutations, enhanced IκB degradation and NF-κB translocation were observed upon TNF-a stimulation. This was accompanied by reduced A20 binding to TRAF2 and RIP1 in the TNFR complex. Immunoprofiling of patients’ cells confirmed enhanced gene expression of NF-κB target genes and overproduction of the proinflammatory cytokines IL-1β, TNF-α, IL-9, IL-17 and IP-10. Patients’ PBMCs also exhibited constitutive NLRP3 inflammasome activation, skewed subsets of pro-inflammatory monocytes, increased gene expression of IL-1β and TNFα in M1 macrophages, and increased Th9 and Th17 differentiation.

## Conclusion

Truncating *TNFAIP3* mutations cause haploinsufficiency of the A20 protein, with upregulation of the NFκB signaling pathway, NLRP3 inflammasome activation, and overproduction of proinflammatory cytokines. Targeted therapies with biologics that inhibit these cytokines may be effective in these patients. This is the first report of a human disease caused by high penetrance germline mutations in *TNFAIP3*.

